# Using Patient Profiles for Sustained Diabetes Management Among People With Type 2 Diabetes

**DOI:** 10.5888/pcd20.220210

**Published:** 2023-03-16

**Authors:** Shang-Jyh Chiou, Yen-Jung Chang, Chih-Dao Chen, Kuomeng Liao, Tung-Sung Tseng

**Affiliations:** 1 Department of Health Care Management, National Taipei University of Nursing and Health Sciences, Taipei City, Taiwan, Republic of China; 2 Department of Health Promotion and Health Education, National Taiwan Normal University, Taipei City, Taiwan, Republic of China; 3 Department of Family Medicine, Far Eastern Memorial Hospital, New Taipei City, Taiwan, Republic of China; 4 Department of Endocrinology and Metabolism, Zhongxiao Branch, Taipei City Hospital, Taipei City, Taiwan, Republic of China; 5 Behavioral and Community Health Sciences, School of Public Health, Louisiana State University Health Sciences Center–New Orleans, New Orleans, Louisiana

## Abstract

**Introduction:**

Our objective was to evaluate the association between patient profiles and sustained diabetes management (SDM) among patients with type 2 diabetes.

**Methods:**

We collected HbA_1c_ values recorded from 2014 through 2020 for 570 patients in a hospital in Taipei, Taiwan, and calculated a standard level based on an HbA_1c_ level less than 7.0% to determine SDM. We used patients’ self-reported data on diabetes self-care behaviors to construct profiles. We used 8 survey items to perform a latent profile analysis with 3 groups (poor management, medication adherence, and good management). After adjusting for other determining factors, we used multiple regression analysis to explore the relationship between patient profiles and SDM.

**Results:**

The good management group demonstrated better SDM than the poor management group (β = 0.183; *P* = .003). Using the most recent HbA_1c_ value and the 7-year average of HbA_1c_ values as the outcome, we found lower HbA_1c_ values in the good management group than in the poor management group (β = −0.216 [*P* = .01] and −0.217 [*P* = .008], respectively).

**Conclusion:**

By using patient profiles, we confirmed a positive relationship between optimal patient behavior in self-care management and SDM. Patients with type 2 diabetes exhibited effective self-care management behavior and engaged in more health care activities, which may have led to better SDM. In promoting patient-centered care, using patient profiles and customized health education materials could improve diabetes care.

**Summary**

**What is already known on this topic?**

Successful diabetes management relies on optimal and acceptable patient behavior. Self-efficacy and self-management are essential factors in diabetes-related health behavior.

**What is added by this report?**

We used the HbA_1c_ level of less than 7.0% to assess the outcome of diabetes control and sustained diabetes management. Latent profile analysis is a novel approach for conceptualizing patient profiles and assessing patient behavior in diabetes control.

**What are the implications for public health practice?**

Using patient health profiles can make medical teams aware of patients’ diabetes care and provide incentives for better patient behaviors. Better behaviors lead to patients’ optimal adherence to diabetes care, and subsequently, better health outcomes.

## Introduction

In the US and around the world, diabetes is a serious public health issue. This metabolic disease is the leading cause of blindness, kidney failure, myocardial infarction, and stroke (1–3). Effective patient self-management of diabetes includes not only working with a medical team but also performing self-care behaviors. In clinical settings, physicians ideally need to consider many clinical or behavioral aspects of diabetes care, such as obesity, comorbidities, age, race, sex, gender, disease duration, life expectancy, and quality of life, to make decisions about treatment (4,5). In reality, clinical practitioners and their patients with diabetes can best manage only a few factors. Using one-size-fits-all guidelines in diabetes care may have limited effects (6,7). Furthermore, in diabetes care, patients who practice self-care management behaviors, which incorporate the core values of self-efficacy and self-regulation (8–10), may improve glycemic control through adherence to the 4 major components of diabetes control: medication adherence, diet, exercise, and the self-monitoring of blood glucose (SMBG). Adequate patient behavior in diabetes care is crucial to reaching optimal health outcomes, and integrated approaches are necessary for a long-lasting effect (11).

Diabetes management is enhanced through behaviors such as exercise, smoking cessation, and eating a healthy diet. Diabetes is a lifetime disease; when patients follow behavioral guidelines inconsistently, they rarely reach optimal goals (12). Integrating diabetes care into their daily lives is often challenging; therefore, patients’ undesirable behaviors are not likely to improve, and appropriate behaviors are unlikely to occur and be maintained over time (13,14). In addition, comprehensive diabetes management plans include factors related to the disease process and patient-management behaviors. To experience optimal quality of life, patients with type 2 diabetes must control their condition by following guidelines on medication adherence, healthy diet, regular physical activity, and blood glucose monitoring, which lowers the risk of macro or micro complications (15,16). Hence, the American Diabetes Association emphasizes the importance of self-care management (17). Therefore, an approach that involves behavioral science to understand patient behavior for alleviating barriers and psychological conditions is imperative in diabetes care.

Some patients can sustain the 4 major components of diabetes control; however, some may only perform efficiently in certain tasks. We are particularly interested in whether different patient subgroups have different levels of risks of future complications. We hypothesized that information from patient profiles classified by multiple behaviors may not only identify groups at high risk of complications but may also be used to customize health education materials. In this patient-centered era, the use of patient profiles and customization of health education materials may be the most efficient way to help patients improve their behaviors.

Traditionally, a variable-oriented approach, such as a regression model, has been used to address the relationship between HbA_1c_ levels and patient behavior–related factors. However, this method cannot easily handle multiple factors simultaneously, thus creating potential for type I errors (18). Additionally, researchers have had to make complicated decisions, such as how many variables to include in a model. To address these challenges, we used latent profile analysis (LPA), a participant-oriented approach that provides rich yet concise information needed to determine a patient’s effort in diabetes control. Physicians may concentrate only on the most recent HbA_1c_ level to categorize a patient’s diabetes control and decide on treatment. For the diabetes control proxy, we used sustained diabetes management (SDM) from the long-term standard HbA_1c_ level.

Using patient profiles and SDM is a novel way of evaluating diabetes management, especially an approach that involves behavioral science, wherein components of profiles in diabetes care are constructed. LPA is an ideal technique to address complex conceptualization (such as patient profiles) for developing typologies based on data (19). The use of patient profiles could help clinical practitioners be aware of patient behaviors in diabetes care; it could also enable health authorities to provide incentives that encourage improved patient behaviors, thus leading to optimal adherence to diabetes care and enhanced health outcomes. Hence, our study aimed to explore the associations between patient profiles and SDM in patients with type 2 diabetes.

## Methods

This study was conducted in the Department of Metabolism at a regional hospital in northern Taipei from November 2019 through May 2020. Trained staff members used structured questionnaires to conduct face-to-face interviews of patients with type 2 diabetes after their medical consultations. Before the interview, all patients provided written informed consent that included use of their biomarker data (HbA_1c_) from the hospital’s health information system. All patients participating in the survey had type 2 diabetes and answered the questionnaire voluntarily; patients with mental disorders or cognitive impairments or who were unable to provide informed consent or participate in the survey were excluded. We included 570 patients in the analysis. After the survey, we compensated participants with an NT$100 gift card. The institutional review board of National Taiwan University and the hospital approved this study.

### Dependent variable: SDM

We obtained the 2014–2020 medical records from the hospital’s health information system of participants who provided written consent. At their physician’s office for a scheduled appointment, participants had an HbA_1c_ test. Each patient had multiple medical records for HbA_1c_ values from 2014 to 2020. We calculated the standard level based on HbA_1c_ criteria (<7.0%). We used the following equation to calculate SDM: SDM = number of HbA_1c_ measurements less than 7.0% divided by the total number of HbA_1c_ events. For example, 1 patient had HbA_1c_ values of 7.1, 7.0, 6.9, 7.1, and 6.7; two of the 5 values were considered standard because they were less than 7.0%. The number of standard values (n = 2) was divided by the number of data points (n = 5) to obtain the SDM: 2/5 = 0.4. Therefore, 0.4 indicated SDM during the study period.

### Independent variable

The questionnaire asked about such characteristics as age, sex, education level, diabetes diagnosis date, and diabetes care–associated variables. Such variables included the self-reported level of health education received by the participants from the medical staff (1–10, with 10 being the highest level); the diabetes management self-efficacy scale in Chinese (DMSES–C); the treatment self-regulation questionnaire on diabetes (TSRQd); a self-report assessment of the self-management of diabetes control pertaining to medication, healthy diet, SMBG, and regular exercise (scale of 1 to 5, with 5 being the highest level of control for all 4 measures); and 2 self-reported items on health status. The original DMSES–C and TSRQd have 20 and 19 items, respectively. They were translated from English into Chinese with acceptable validity and reliability. After consulting with the translation team and experts in using the DMSES-C and TSRQd, we used shorter versions of the DMSES-C (shortened to 11 items) and the TSRQd (shortened to 15 items), which excluded some items from the 2 questionnaires as described elsewhere (20). On the basis of the original design (21,22), the user can categorize the TSRQd into 2 dimensions, namely autonomous regulatory style (which involves conducting an activity for the enjoyment inherent in engaging in the behavior itself) and controlled regulatory style (which includes behavior motivated by contingencies not inherent to the activity itself), which we defined as TSRQd–A and TSRQd–C, respectively. The questionnaire asked patients the following health status–related question: “As compared to the past 12 months, how would you evaluate your current health status: better, neutral, or worse?” We also scored their self-reported health status from 0 to 100, with 100 indicating the best.

### Statistical analysis

In the last 2 decades, LPA has been used extensively in the social sciences; additionally, it has been applied in the medical field to cluster individuals into subgroups and unveil hidden patterns of association, such as different risk groups or social support levels. We used 8 survey items on patients’ self-report of diabetes self-care behaviors for constructing patient profiles in the LPA. Eight items included scores for self-assessment in health education; medication self-management, healthy diet, SMBG, regular exercise; self-efficacy (DMSES–C), and self-regulation evaluation (TSRQd–A and TSRQd–C).

After comparing Akaike information criterion, Bayesian information criterion, and entropy at 3, 4, and 5 class levels (Table 1), we decided to use the following 3 groups in the LPA model: poor management, medication adherence, and good management (Figure). Of the 570 patients in our sample, 6% (n = 35) were in the poor management group, 21% (n = 117) were in the medication adherence group, and 73% (n = 418) were in the good management group (Table 2). The good management group had the highest mean values for all survey items. In contrast, the medication adherence group had the lowest mean values for all survey items, except medication self-management. The poor management group self-reported a slightly higher mean value than the medication adherence group for most items.

**Table 1:** Information Criteria for Patient Profiles Derived From Latent Profile Analysis in Study on Sustained Diabetes Management Among People With Type 2 Diabetes, Taiwan, 2014–2020

Criteria	3 Groups	4 Groups	5 Groups
Akaike Information Criterion	18,924.5	18,780.6	18,608.8
Bayesian Information Criterion	19,069.2	18,967.5	18,834.8
Adjusted Bayesian Information Criterion	18,961.3	18,831.0	18,669.7
Entropy	0.853	0.826	0.947

**Figure:**
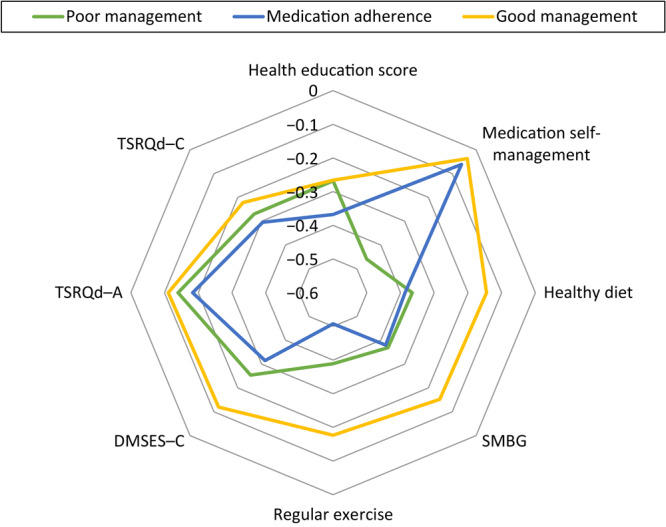
Radar chart displaying the percentile of each indicator from the response frontier in different patient profiles. The response frontier was used to calculate the deviation from the highest score for every item caused by wider variations in the item scale ranges. For example, health education scores in poor management, medication adherence, and good management are 7.3, 6.3. and 7.3, respectively; therefore, the response frontier of health education score is (10 − 7.3)/10 = 0.27; (10 − 6.3)/10 = 0.37, and (10 − 7.3/10) = 0.27, respectively. The outer ring (the good management group) depicts better performance than the inner rings (the poor management group and the medication adherence group) in the 8 items used for the latent profile analysis. Abbreviations: SMBG, self-monitoring of blood glucose; DMSES–C, diabetes management self-efficacy scale (Chinese version); TSRQd–A, treatment self-regulation questionnaire on diabetes–autonomous regulatory style; TSRQd-C, treatment self-regulation questionnaire on diabetes–controlled regulatory style.

**Table:** 

Survey item	Patient score			Response frontier		
	Poor management	Medication adherence	Good management	Poor management	Medication adherence	Good management
Health education score	7.3	6.3	7.3	−0.27	−0.37	−0.27
Medication self-management	2.7	4.7	4.8	−0.46	−0.06	−0.04
Healthy diet	3.2	3.1	4.3	−0.37	−0.39	−0.14
SMBG	3.2	3.1	4.2	−0.37	−0.38	−0.15
Regular exercise	3.1	2.5	4.1	−0.39	−0.51	−0.18
DMSES–C	41.0	37.7	48.4	−0.26	−0.31	−0.12
TSRQd–A	34.4	32.7	35.6	−0.14	−0.18	−0.11
TSRQd–C	25.6	24.4	27.2	−0.27	−0.30	−0.22

**Table 2:** Results of Survey Among 570 Patients With Type 2 Diabetes, Taiwan, 2014–2020

Survey item	Mean score	Poor management (n = 35)	Medication adherence (n = 117)	Good management (n = 418)
Health education^a^	7.1	7.3	6.3	7.3
Medication self-management^b^	4.7	2.7	4.7	4.8
Healthy diet^b^	3.9	3.2	3.1	4.3
Self-monitoring of blood glucose^b^	3.9	3.2	3.1	4.2
Regular exercise^b^	3.7	3.1	2.5	4.1
Diabetes management self-efficacy scale (Chinese version)^c^	45.6	41.0	37.7	48.4
Treatment self-regulation questionnaire on diabetes–autonomous regulatory style^d^	34.8	34.4	32.7	35.6
Treatment self-regulation questionnaire on diabetes–controlled regulatory style^e^	26.5	25.6	24.3	27.2

a Self-reported level of health education received by the participants from the medical staff (1–10, with 10 being the highest level).

b Scale of 1 to 5, with 5 being the highest level of control.

c Scale of 1 to 5, with 55 indicating the highest score (11 items).

d Conducting an activity for the enjoyment inherent in engaging in the behavior itself. Scale of 1 to 5, with 40 indicating the highest score (8 items).

e Behavior motivated by contingencies not inherent to the activity itself. Scale of 1 to 5, with 35 indicating the highest score (7 items).

We summarized information on patient profiles derived from the LPA. We used χ^2^ tests and 1-way analysis of variance to initially evaluate patient demographic data and the distribution of survey item responses by LPA subgroup. We used multiple regression analysis to estimate the likelihood of SDM by LPA subgroup with the other determining factors (Model 1). Considering that medical personnel were familiar with the HbA_1c_ value as a marker for diabetes control, we used the most recent HbA_1c_ value (Model 2) and the 7-year average of HbA_1c_ values (Model 3) to conduct the regression model again for a sensitivity analysis. Subsequently, with the same determining factors, we compared the results of Models 2 and 3 with those of Model 1. Education, health status, and the LPA subgroups were transformed into dummy variables in the regression models. We used SAS version 9.3.1 (SAS Institute, Inc) and SPSS 20.0 (IBM Corporation) to analyze all data. The significance level was set at .05.

## Results

The good management group had the oldest mean age (63.1 y), the highest scores for health status (76.9 vs 70.1 [poor management group] and 69.5 [medication adherence group]), the longest diabetes duration (11.9 y vs 9.3 y [poor management group] and 10.3 y [medication adherence group]), and the highest proportion of patients with a standard HbA_1c_ rate of ≥0.5 (70.1% vs 51.3% [poor management group] and 55.7% [medication adherence group]) (Table 3). Moreover, the good management group had better behaviors for diabetes control (Table 2) than the other 2 groups. Differences in sex, education level, and health status were not significant.

**Table 3:** Characteristics, Health Status, Diabetes Duration, and HbA_1c_ Standard Level Rate Among Patient Profile Groups in Study on Sustained Diabetes Management Among People With Type 2 Diabetes (N = 570), Taiwan, 2014–2020^a^

Characteristic	Poor management group (n = 35)	Medication adherence group (n = 117)	Good management group (n = 418)	*P* value^b^
**Sex**				
Male	21 (60.0)	70 (59.8)	253 (60.5)	.99
Female	14 (40.0)	47 (40.2)	165 (39.5)	
**Age, mean (SD), y**	55.3 (12.4)	58.3 (14.4)	63.1 (11.9)	<.001
**Education**				
Primary school	4 (11.4)	22 (18.8)	88 (21.1)	.65
Junior high school	4 (11.4)	18 (15.4)	52 (12.4)	
Senior high school	14 (40.0)	31 (26.5)	116 (27.8)	
College and above	13 (37.1)	46 (39.3)	162 (38.8)	
**Health status compared with previous 12 months**				
Worse	6 (17.1)	20 (17.1)	65 (15.6)	.94
Neutral	20 (57.1)	63 (53.8)	220 (52.8)	
Better	9 (25.7)	34 (29.1)	132 (31.7)	
**Health status score, mean (SD)^c^**	69.5 (12.0)	70.1 (13.4)	76.9 (11.9)	<.001
**Diabetes duration, mean (SD), y**	9.3 (6.7)	10.3 (7.3)	11.9 (7.8)	<.001
**HbA_1c_ standard level**				
<0.5	14 (48.3)	43 (44.3)	109 (29.9)	.007
≥0.5	15 (51.3)	54 (55.7)	256 (70.1)	

Abbreviation: HbA_1c_, glycated hemoglobin A_1c_.

a Values are number (percentage) unless otherwise indicated.

b Determined by χ^2^ test and analysis of variance.

c Scored from 0 to 100, with 100 indicating best health.

In Model 1, the good management group and medication adherence group were more likely than the poor management group to achieve better SDM (β = 0.183 [*P* = .003] and 0.120 [*P* = .07], respectively) (Table 4). Patients with a longer diabetes duration had lower SDM (β = −0.015; *P* < .001). In Models 2 and 3, which used the most recent HbA_1c_ value and the 7-year average for HbA_1c_ values, patients with a longer diabetes duration had higher HbA_1c_ values (Model 2 β = 0.184, *P* < .001; Model 3 β = 0.208; *P* < .001). Conversely, we found lower HbA_1c_ values among older patients (Model 2 β = −0.165, *P* = .002; Model 3 β = −0.274, *P* < .001) and the good management group (Model 2 β = −0.216, *P* = .01; Model 3 β = −0.217, *P* = .008).

**Table 4:** Comparison of Sustained Diabetes Management, Most Recent HbA_1c_ Value, and 7-Year Average of HbA_1c_ Value With Patient Profiles Derived From Latent Profile Analysis

Characteristic	Model 1,^a^ β (95% CI) [*P* value]	Model 2,^b^ standardized β (*P* value)	Model 3,^c^ standardized β (*P* value)
**Male sex**	0.045 (−0.014 to 0.103) [.13]	0.030 (.52)	−0.005 (.91)
**Age**	0.008 (0.005 to 0.011) [<.001]	−0.165 (.002)	−0.274 (<.001)
**Education**			
Primary school	Reference	Reference	Reference
Junior high school	0.030 (−0.070 to 0.131) [.56]	0.045 (.41)	0.031 (.55)
Senior high school	0.132 (0.047 to 0.217) [.002]	−0.015 (.81)	−0.113 (.06)
College and above	0.154 (0.069 to 0.239) [<.001]	−0.071 (.28)	−0.179 (.005)
**Health status compared with previous 12 months**			
Worse	Reference	Reference	Reference
Neutral vs worse	0.036 (−0.055 to 0.126) [.44]	−0.036 (.59)	−0.052 (.42)
Better vs worse	0.093 (0.011 to 0.174) [.03]	−0.114 (.08)	−0.143 (.02)
**Health status score**	0.001 (−0.001 to 0.004) [.33]	−0.031 (.52)	−0.068 (.15)
**Diabetes duration**	−0.015 (−0.018 to −0.011) [<.001]	0.184 (<.001)	0.208 (<.001)
**Patient profile group**			
Poor management	Reference	Reference	Reference
Medication adherence	0.120 (−0.009 to 0.250) [.07]	−0.136 (.10)	−0.155 (.054)
Good management	0.183 (0.062 to 0.303) [.003]	−0.216 (.01)	−0.217 (.008)

a Model 1 used sustained diabetes management, standard HbA_1c_ level rates during 7-year study period.

b Model 2 used the most recent HbA_1c_ value.

c Model 3 used the 7-year average for HbA_1c_ values.

## Discussion

Our study used patient profiles to show that enhanced self-assessment in diabetes care, including diet, medication, exercise, and SMBG, self-efficacy, and self-regulation, may lead to improved SDM. The moderate association between patient profiles and SDM demonstrates a novel way to manage the manifest indicator of diabetes control from multiple years and classify patient behaviors in summary profiles derived from multiple dimensions. Apparently, when patients with type 2 diabetes had better self-care behaviors, they had a greater likelihood of having acceptable HbA_1c_ levels (defined by a 7.0% cut point). In addition, patients with more motivation to engage in health promotion and health care behaviors (autonomous or self-determined) had better outcomes in SDM. Our results are consistent with the results of previous studies (23,24). The main goal of the 4 major components of diabetes control (diet, medication, exercise, and SMBG) is to maintain HbA_1c_ at an optimal level to reduce the risk of complications, such as retinopathy, nephropathy, neuropathy, and stroke (25). Moreover, according to social cognitive and self-determination theory, patients believe they can execute the behaviors necessary for producing and maintaining performance outcomes in accordance with the demands of diabetes care (26,27).

The Association of Diabetes Care & Education Specialists has provided an evidence-based model to help patients improve the behaviors necessary for diabetes self-management and increase their self-efficacy toward such self-care activities (28). Although patients themselves play an important role in diabetes management, they may not change all their self-management behaviors to align with suggested standards. For example, low- and middle-income individuals have exhibited inadequate self-care behaviors because of the extensive dietary restrictions required and the suggestions for SMBG (29). Previous studies listed unsurprising barriers to adequate self-care behaviors, such as a lack of motivation or inadequate knowledge and skills (30,31). The integration of self-efficacy (28,32), self-regulation, and other factors with adherence to self-care behaviors among patients with diabetes could be complicated for the health education teams who are making immediate decisions in a limited amount of time, especially in a clinical setting. Therefore, we used 8 survey items to depict different patient profiles; this succinct questionnaire may help health care providers capture data on patient characteristics and their diabetes-related self-care behaviors.

Patients in the poor management group had the lowest values for most self-care behaviors; however, they had slightly better values for self-regulation and health education. These patients were defined as having ineffective management. Although patients in the medication adherence group performed poorly in most self-care behaviors, they had the highest score in medication adherence and were described as demonstrating medication adherence. Patients in the good management group performed appropriately in all self-care behaviors.

The good management group had better SDM than the other 2 groups, indicating that diabetes management should include medication as well as a healthy diet and physical activity. However, in our research, the use of patient profiles derived from LPA insufficiently captured the characteristics that reflect different patient behaviors. For example, the average scores of healthy diet and SMBG in the poor management and medication adherence groups were very close. Possible explanations include our small sample size, the limited number of dimensions, and the homogeneity of the items in the self-care evaluation to extract the subgroup information from the LPA model. Further studies should either adopt the diversity of patient behaviors based on the conception of behavioral science for validation or use a large database to create patient health profiles from LPA. Such information could help physicians analyze patient behaviors in diabetes care and develop customized diabetes control plans. Nonetheless, our novel method of using patient profiles in a clinical setting is beneficial. By evaluating patient behaviors through the use of limited questions in the decision-making process, physicians were not only able to review biomarker tendencies but also obtain a snapshot of behaviors from patient profiles. Thus, this approach helped them create diabetes management suggestions that their patients can understand.

The American Diabetes Association suggests that health care providers offer diabetes self-management education and support (DSMES) that considers a patient’s confidence and self-efficacy behaviors as well as family and social support (14). However, many contributing factors can hinder behavior change in diabetes management, ranging from motivation, skills, and resources to social support and the environment. Being able to address all possible factors is ideal; however, in doing so, the enormous complexity of diabetes and the one-size-fits-all behavior change can be overwhelming for patients. Thus, tailored strategies that can help them overcome modifiable barriers are needed. By using patient profiles, we found that most (>70%) patients achieved favorable measures of diabetes control. However, the remaining participants relied on medication to control their diabetes and focused on health education to change their diet, exercise, and SMBG behaviors. Although a balanced method for diabetes management is the optimal approach, we can also consider using an easy-scale survey of the major components of self-care diabetes behavior. Through such an investigation, health education teams can provide additional resources to help patients overcome barriers to improving diabetes self-management.

Medical teams may be more familiar with appraising biomarkers, such as the most recent HbA_1c_ values in the medical record or the fluctuation of values over 3 to 5 years, than with assessing patients’ self-reported self-management. Interestingly, using either the most recent HbA_1c_ value or the 7-year average of the HbA_1c_ values leads to the same conclusion and demonstrates the potential of applying patient profiles in diabetes care. A patient-oriented approach, such as LPA, can be a better alternative for understanding diabetes management behavior as a totally functioning, not separate, variable, in terms of whole-system properties. The patient-oriented approach used in our study produced a condensed summary from a single categorical variable, as good at predicting an outcome as the original variables, and it also bypassed the difficulty of testing the interaction on empirical data in a variable-oriented approach. Health care providers may use such information obtained from patient profiles to help patients adjust their lifestyles according to customized suggestions. However, we need additional evidence to demonstrate and develop a practical approach (eg, a checklist) in clinical settings for diabetes management.

### Limitations

This study has several limitations. First, although the use of patient profiles is a novel approach, it might not deal perfectly with multiple factors together; this shortcoming would be especially applicable when some factors are excessively homogenous in the population of interest. Our study used 8 items in the LPA model, including self-assessment in health education, self-management of medication, healthy diet, SMBG, regular exercise, self-efficacy, and self-regulation. Additionally, risk behaviors such as alcohol consumption, smoking, and family and environmental factors should be considered. Second, selection bias may have been possible. Compared with patients who did not participate, survey participants may have been more aware of their diabetes condition and more willing to comply with suggested diabetes care behaviors. We could have considered using a randomized trial or including all patients with type 2 diabetes in the hospital’s Department of Metabolism to obtain richer information. Third, the use of the standard HbA_1c_ level is not common in clinical settings; furthermore, its accuracy has not been validated in diabetes care management. Finally, our survey did not consider several important factors, such as motivation or health literacy; thus, we did not examine their function in patient profiles. Our study had information on education level, which is strongly associated with literacy, and the TSRQd can be used for measuring motivation. However, because of insufficient measurements of these factors, we would be cautious about extending our explanations about them. Furthermore, we realize that diverse constructions in patient profiles may lead to different concepts of patient behaviors in diabetes management; it may be essential for future studies to consider multidisciplinary dimensions in patient behaviors to develop useful tools in diabetes care.

### Conclusions

Using patient profiles derived from LPA confirmed the positive relationship between optimal patient behaviors in self-care management and SDM. Patients with type 2 diabetes exhibited good self-care management behaviors and confidence in these behaviors; moreover, greater engagement in health care behaviors may lead to improved SDM. However, additional information is required to validate the application of patient profiles in diabetes care in clinical settings. In promoting patient-centered care, the use of patient profiles with customized health education materials is a worthwhile approach to diabetes care.
